# Isolation and Multiple Differentiation of Rat Pericardial Fluid Cells

**DOI:** 10.3389/fcell.2021.614826

**Published:** 2021-02-11

**Authors:** Ying Sun, Yan Wang, Zongjin Li, Zhikun Guo

**Affiliations:** ^1^Henan Key Laboratory of Medical Tissue Regeneration, Xinxiang Medical University, Xinxiang, China; ^2^Department of Cardiology, Zhengzhou Seventh People’s Hospital, Zhengzhou, China

**Keywords:** CD90, CD44, CD29, CD45, cell differentiation, pericardial fluid

## Abstract

**Objective:**

The aim of the present study is to isolate and analyze the characterization of pericardial fluid cells (PFCs) from rat and provides a morphological basis for the basic research and clinical application of PFCs.

**Methods:**

After aseptic thoracotomy was performed, normal saline was injected into the pericardial cavity of 50 adult Sprague–Dawley rats. The mixture of diluted pericardial fluid was extracted, centrifuged, and cultured. The cell morphology of different generations in the pericardial fluid was observed on an inverted microscope. The expression levels of CD44, CD29, CD90, and pan-hematopoietic marker CD45 were analyzed via flow cytometry. The third-generation cells were used for osteogenic, adipogenic, and cardiac differentiation.

**Results:**

PFCs were successfully isolated and subcultured. PFCs were predominantly circular in shape after 24 h of culture. Following subculture for 3 days, the cells demonstrated a spindle shape. The rat pericardial fluid contains cell populations with uniform morphology, good growth state, and strong proliferation ability. Flow cytometry results showed that CD29 (100%) and CD90 (99.3%) were positively expressed, whereas CD45 (0.30%) and CD44 (0.48%) were negatively expressed. The PFCs could differentiate into osteoblasts and adipocytes after being induced. Cardiac differentiation was also confirmed by cardiac troponin T (cTnT) and α-sarcomeric actin (α-SA) staining.

**Conclusion:**

This study revealed that a subpopulation of cells was isolated from pericardial fluid, which exhibited progenitor cell features and multiple differentiation potency. PFCs could serve as an alternative cell source for myocardial tissue repair, engineering, and reconstruction.

## Introduction

The pericardium consists of two layers, the outer fibrous layer and the inner serous layer (Buoro et al., 2020). The pericardial fluid is a serous fluid that exists between the serous pericardial viscera and the parietal layer. This fluid also serves as a cushion and reduces friction between the central viscera and the wall during the cardiac cycle. The normal pericardial fluid volume of adult human is approximately 50 mL (Phelan et al., 2015). The pericardium protects the heart by preventing aberrant motion, excessive dilation due to acute volume overload, and the spread of infections from other organs (Beltrami et al., 2017; Sahoo et al., 2017). Therefore, pericardial mesothelial cells could also synthesize and secrete certain vascular active substances into the pericardial cavity. Recent study revealed that mesothelial cells are abundant in pericardial fluid, which included other kinds of cells, such as neutrophils, lymphocytes, monocytes, and macrophages (Buoro et al., 2020). In the case of disease, the composition of the cellular fluid could be changed. High concentrations of vascular-derived growth factor accumulate in the pericardial fluid of patients with ischemic heart disease. This phenomenon may have a self-protective mechanism against myocardial ischemia (Fujita et al., 2001). Studies have shown that stem cells exist in various body fluids. The mesenchymal progenitor cells in the synovial fluid of patients with arthritis possess multiple mesenchymal differentiation potentials (Jones et al., 2004). Cells isolated from urine could differentiate into several bladder cell lineages expressing markers of urothelial, endothelial, and interstitial cells (Zhang et al., 2008). These cells are currently being investigated for tissue engineering applications. In the present study, flow cytometry, cell culture, and induction were used to detect the cellular components and differentiation potential of rat pericardial fluid.

## Materials and Methods

### Experimental Animals

Fifty adult male Sprague–Dawley rats between 8 and 12 weeks old with an average body weight of 120 ± 20 g were housed with a 12-h light/dark cycle (lights on from 7:00 to 19:00) at constant temperature (25°C). All animal protocols were conducted in accordance with the guidelines of the Ministry of Science and Technology of the People’s Republic of China [(2006)398] and approved by the Animal Care Committee of Xinxiang Medical University (No. 030032).

### Extraction, Cell Isolation, and Culture of Rat Pericardial Fluid Cells

The thorax of healthy adult rats was opened in the middle of the sternum under sterile conditions after anesthesia and disinfection. The hooks were fixed to expose the pericardium. A 2 mm incision was cut near the pericardial fat, and 0.5 mL of normal saline was drawn with a trocar and injected into the pericardial cavity. The pericardial fluid was diluted, and a tube of cell suspension was taken from the pericardial fluid of approximately five rats. After the suspension was filtered, pericardial fluid cells (PFCs) were collected. The PF samples were centrifuged at 4°C (1200 r/min, 5 min), the cell pellets were washed with phosphate-buffered saline, and the supernatant was discarded. The cells were resuspended in complete medium (low-glucose DMEM, 30% fetal bovine serum, 100 U/mL of penicillin, and 100 U/mL of streptomycin) and inoculated for 24 h. On day 3, fluid exchange was observed, and the non-adherent cells were discarded. The fluid was changed every 3 days thereafter. When the cells were approximately 70–80% of the bottom of the bottle, they were digested with 0.25% protease (containing 0.02% EDTA, 0.04 mL/cm^2^) and passaged. The passage ratio was 1:2.

### Flow Cytometric Identification

P3 cells were seeded in a T75 cell culture flask. After 3 days, the old culture solution was discarded, and the cells were thoroughly washed to remove the suspended cells. The cells were digested with 0.25% trypsin (containing 0.02% EDTA, 0.04 mL/cm^2^), resuspended, and counted in PBS. Fluorescence-labeled CD45-FITC (BioLegend, 1:100, United States), CD90-PE (BioLegend, 1:100, United States), CD44-PE (eBioscience, 1:100, United States), and CD29-FITC (BioLegend, 1:100, United States) monoclonal mouse antibodies were mixed, stored at 4°C in the dark for 30 min, and centrifuged at 1200 r/min for 5 min. The supernatant was discarded, and the excess antibodies were eluted three times. Isotype-identical antibodies were served as controls (BD Pharmingen). Detection was performed using the BD FACSCanto flow cytometry system (BD Corporation, United States).

### Differentiation of Induced and Cultured PFCs Into Osteoblasts

The isolated P2 cells were seeded in a 24-well plate at a density of 1.2 × 10^6^/cm^2^ and divided into six experimental groups (*n* = 3) and one control group (*n* = 3). When approximately 60–70% of the cells adhered to the other cells, the culture medium was replaced with bone culture medium (high-sugar DMEM containing 10% fetal bovine serum, 0.1 μmol of dexamethasone, 20 mmol/L of β-phosphate glycerol, and 50 μmol/L of ascorbic acid). The control group was cultured in complete medium. Cell morphology was regularly observed on an inverted microscope. After approximately 7 days, the protruding cells were retracted and connected tightly to form cell clusters. The experiment was repeated three times. The culture medium in each group was changed every 3 days. After 21 days of induction, the cells were fixed with paraformaldehyde for Alizarin red staining as previously reported (Zhang et al., 2008).

### Differentiation of Induced and Cultured PFCs Into Adipocytes

The isolated P2 cells were seeded in a 24-well plate at a density of 1.2 × 10^6^/cm^2^ and divided into six experimental groups (*n* = 3) and one control group (*n* = 3). When approximately 60–70% of the cells adhered to the other cells, the culture medium was replaced with an adipocyte-induction solution (low-sugar complete medium containing 1 μmol/L of dexamethasone, 0.01 g/L of insulin, 200 μmol/L of indomethacin, and 0.5 of mmol/L isobutyl methyl xanthine). The control group was cultured in complete medium, and the cells were cultured at 37°C in 5% CO_2_ for 21 days, during which the solution was completely replaced every 3 days. Cell morphology was regularly observed on an inverted microscope. Some cells showed increased dendritic bifurcations after approximately 7 days. The experiment was repeated three times. Subsequently, the adipocytes were washed with PBS, fixed with paraformaldehyde for 20 min, stained with Oil Red O solution for 20 min at room temperature, rinsed twice with 60% isopropanol, and washed again with PBS, followed by observation under the microscope and image acquisition (Wei et al., 2019; Hou et al., 2020).

### Multiple Differentiation of PFCs

The isolated P2 cells were seeded in a 24-well plate at a density of 1.2 × 10^6^/cm^2^ and divided into six experimental groups (*n* = 3) and one control group (*n* = 3). When approximately 60–70% of the cells adhered to the cell, the culture medium was replaced with myocardial culture medium (DMEM low-sugar medium containing 10% fetal bovine serum with 5 μmol of E61541, 5 μmol of phenylcyclopropylamine, 10 μmol of CHIR99021, 10 μmol of forskolin, 1 μmol of dorsomorphin, and 2 μmol of IWR-1). The control group was cultured in complete medium and co-cultured for 7 days. Cell morphology was observed on an inverted microscope every 3 days. After 3 days, some cells exhibited increased dendritic protrusions, and all cells showed concentric circles or clumps. Afterward, the culture medium was changed every 3 days. After 7 days of induction, cells were fixed with 4% paraformaldehyde for 20 min and then immersed in PBS three times for 5 min each time. Subsequently, 0.3% Triton X-100 was added to the permeable membrane for 20 min. The cells were immersed with PBS three times for 5 min each time and added with goat serum blocker (Bosterbio) for 60 min. The blocking solution was aspirated without washing, and primary anti-mouse polyclonal antibody cTnT (Abcam, 1:200) mix with/without Rabbit polyclonal to sarcomeric alpha actinin antibody (α-SA; Abcam, 1:500, United States) was added at 4°C overnight and used the next day. The primary antibody was washed with PBS three times. Afterward, secondary antibody Cy3/Alexa Fluor 488 goat anti-mouse/rabbit (Biyuntian, 1:500, United States) was added, and the mixture was incubated at room temperature for 2–4 h. Finally, nuclei were counter stained with DAPI.

Quantification of positive induced cells was performed by counting adipocytes (at least five lipid droplets observed in a cell), osteoblasts (at least lager than 5 μm calcified nodules), and cardiac-like cells (red and green) from nine randomly selected fields (three fields/well). Mean positive cell numbers were calculated for each group. ImageJ software was used to calculate cell numbers.

### Statistical Analysis

All results presented are from at least three independent experiments for each condition. Data are expressed as scatter plots with mean ± standard deviation (SD). Statistical analysis was performed by one- or two-way ANOVA using GraphPad (PRISM software). Differences were considered statistically significant at *P* < 0.05.

## Results

### Morphological Characteristics of Rat Primary PFCs

**FIGURE 1:**
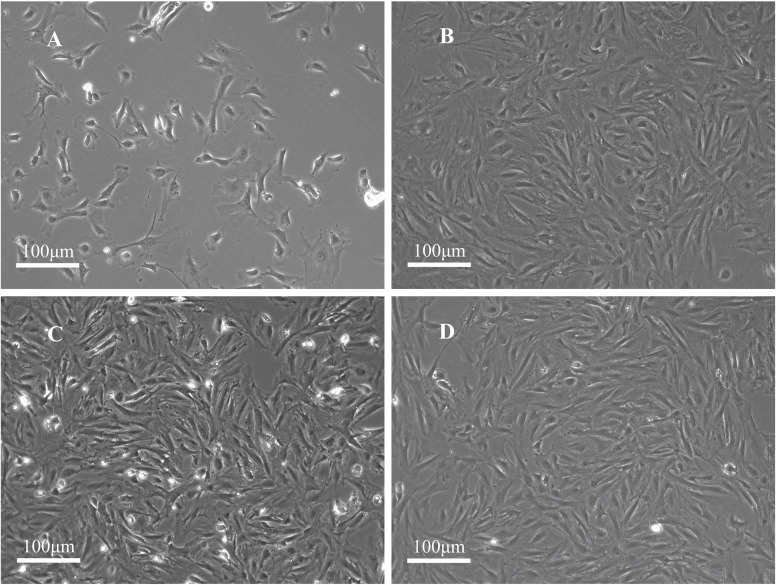
Morphology of rat PFCs. **(A)** Primary PFCs were cultured for 1 day and circular-shaped cells of uniform size. **(B)** PFCs at the first passage were cultured for 3 days. **(C)** PFCs at the second passage were cultured for 3 days. **(D)** PFCs at the third passage were cultured for 3 days. PFCs were spindle-shaped (fibroblast-like cells) in first, second, and third passages after cultured for 3 days. Scale bar: 100 μm.

The PFCs were attached to the surface of the plate and predominantly circular in shape with uniform size. After the PFCs were cultured for 24 h, they turned into spindle or star shaped. The nucleus was located at the center of the cell. During the growth process, the nucleus was gradually vortexed. After the cells were passed, they gradually shrank, and many of them exhibited a polygonal angle (Figures 1A,B). They grew rapidly at the first, second, and third passages, reaching 100% confluence in 2–3 days. The cell morphology was uniform, mainly polygonal cells (fibroblast-like cells, Figure 1C), which is a typical morphology of PFCs (Figure 1D).

### Flow Cytometry of Identified Surface Markers

**FIGURE 2:**
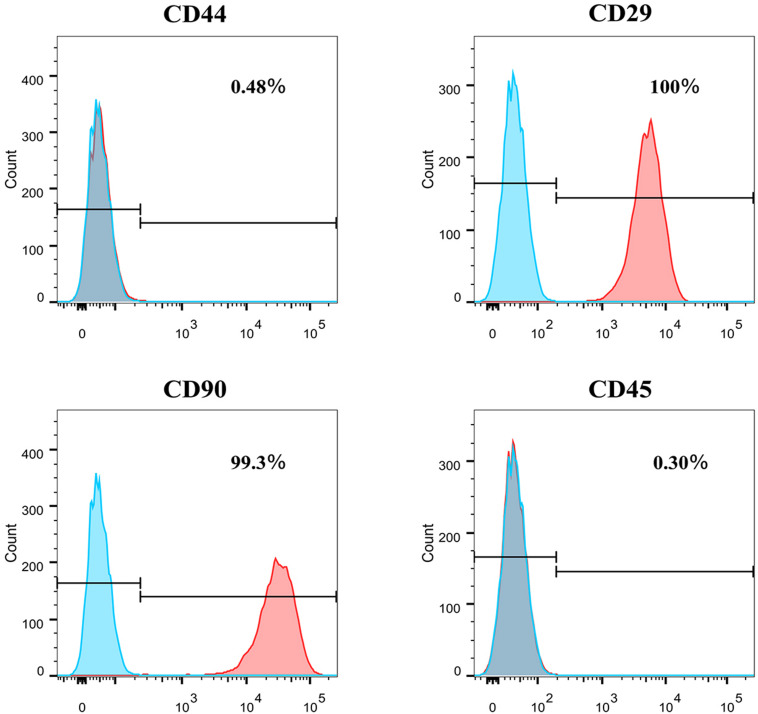
The surface markers expression of CD44, CD29, CD90, and CD45 of P3 PFCs of rats by flow cytometry.

Flow cytometry was used to assess the marker expression. FACS revealed that the PFCs of third passage were positive for some surface markers, which is a characteristic of other various stems/progenitor populations. As shown in Figure 2, these cells were CD90 and CD29 positive, markers for mesenchymal stem cells (MSCs) (Zhao et al., 2019), but negative of MSCs marker CD44. Moreover, this population cells were negative for pan-hematopoietic cell markers CD45, indicating that these cells were not hematopoietic derived cells and also different from MSCs. Immunofluorescence staining showed the same result with flow cytometry (Supplementary Figure 1).

### Multipotent Differentiation of PFCs *in vitro*

**FIGURE 3:**
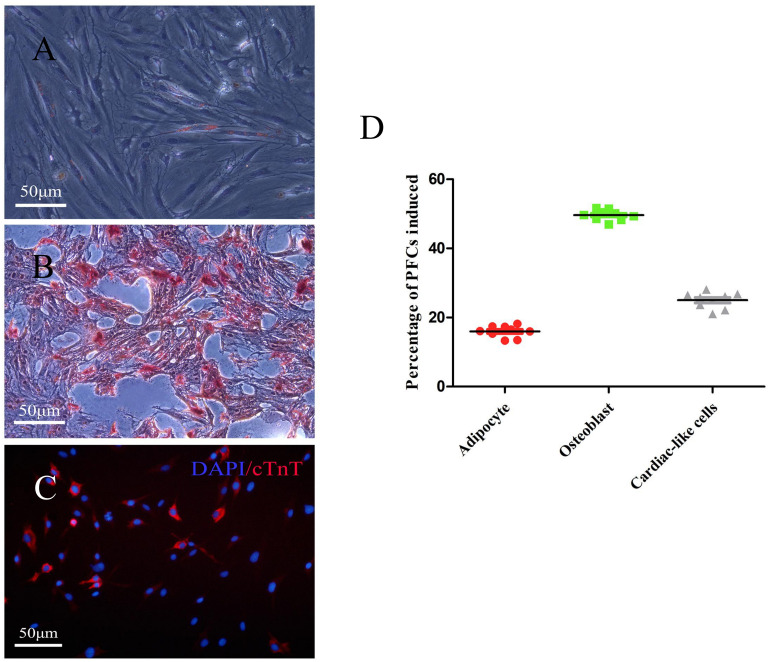
Representative images showing differentiation of PFCs. **(A)** Adipogenic differentiation with oil-red O staining. **(B)** Osteogenic differentiation with Alizarin red staining. **(C)** Cardiac differentiation with cTnT staining. Scale bar: 50 μm. **(D)** The relative percentage of PFCs induced is shown as a scatter plot. Lines in scatter plot represent individual data points with indicated mean ± SD.

To investigate the multilineage differentiation of PFCs, osteogenic, adipogenic, and cardiac differentiation were done. The accumulation of lipid vacuoles during adipogenic induction was observed in PFC cultures. Oil Red O staining showed intracellular lipid red staining (Figure 3A). In contrast to the negative control, all PFCs were grown in osteogenic medium, the number of cells was decreased, and the cell volume was gradually increased. Small nodules and granules were appeared in the cells, and floating matter was precipitated. Alizarin red staining showed red clumps or strip-shaped precipitates with obvious calcified nodules (Figure 3B). To determine the cardiac differentiation of PFCs, PFCs were cultured in cardiomyocyte differentiation medium and immunofluorescent staining confirmed cardiomyocyte marker cTnT expression after 7 days (Figure 3C). In the media without differentiation supplements, osteogenic, adipogenic, and cardiac differentiation was not detected (Supplementary Figure 2). Quantitative analysis of the multipotent differentiation of PFCs showed the percentage of differentiation toward adipocytes, osteoblasts, and cardiac-like cells (Figure 3D).

**FIGURE 4:**
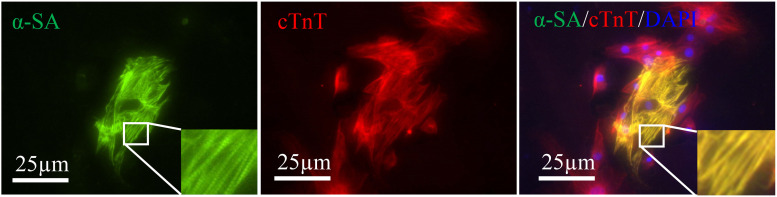
Induced cardiac-like cells expressed cTnT (red) together with α-SA (green). Sarcomeres are magnified and highlighted in rectangle. Scale bar: 25 μm.

Alpha-sarcomeric actin (α-SA) proteins represent the structural building blocks of heart muscle (van der Velden and Stienen, 2019), which are essential for contraction and relaxation. Double staining revealed the α-SA expression in some cTnT positive cells (Figure 4). However, no beating cells were detected in the present study.

## Discussion

Hematologic and biochemical analyses of the pericardial fluids showed that the concentration of small molecules corresponded to a plasma ultrafiltrate (Buoro et al., 2020). LDH and protein levels are higher than plasma ultrafiltrate (Ben-Horin et al., 2005). The pericardial fluid could reflect the immediate changes in the molecular environment around the myocardium after ischemia; it could be used as an important clinical research object (Fujita et al., 2001). In a recent study, the plasma exosomes of pericardial fluid were digested with proteinase K and RNase to prove that they contain at least 16 miRNAs. Pericardial fluid exosomes can improve the survival, proliferation, and angiogenesis of endothelial cells cultured *in vitro* (Beltrami et al., 2017). Soluble mediators and regulatory factors can be released in the pericardial fluid, thereby inducing the growth of coronary artery blood vessel after myocardial infarction (Limana et al., 2007). After myocardial infarction, the exosomes in the pericardial fluid can induce endothelial–mesenchymal transition of the epicardium, enhance arteriogenesis, and reduce cardiomyocyte apoptosis. Pericardial fluid could also be used as a signal to evaluate cardiac pathological changes (Xiang et al., 2013; Foglio et al., 2015). A large amount of T cell infiltration and increased cytokines could be observed in the pericardial fluid of patients with chronic heart failure, leading to changes in the myocardial structural matrix and cause myocardial cell apoptosis that results in inflammation, which reflects T cell activation (Iskandar et al., 2017). The tissue origins of PFCs remain unclear. However, these findings suggested that pericardial fluid serves as a lubricating agent during the cardiac cycle and has many known and unknown physiological effects.

The majority of body fluids contain stem cells, and these cells are important for tissue regeneration and repair (Jones et al., 2004; Zhang et al., 2008; Ji et al., 2017). This observation serves as a reminder whether there are cellular components similar to stem cells in pericardial fluid. In the present study, the flow cytometry results showed that almost all cells expressed CD29 (100%) and CD90 (99.3%), proving that rat PFCs are MSCs like stem cells. Some cells in the rat pericardial fluid could differentiate into adipogenesis, osteogenesis, and cardiomyocytes via induction, thereby reflecting the characteristics of these cells as stem cells. This study was the first to prove that the PFCs from pericardial fluid contain stem cells that are capable of proliferation *in vitro*, and their multipotentiality was investigated. These stem-cell characteristics of PFCs may play a role in the regeneration of myocardial or pericardial tissue; they may also participate in the formation of pericardial fluid components through their autocrine and paracrine effects, which in turn affect the metabolism of surrounding tissues. The CD90 molecule is a cell surface glycoprotein anchored by glycophosphatidylinositol. CD90 has a single V-like immunoglobulin domain and is considered to be a surrogate marker for stem cells, such as hematopoietic stem cells (HSCs). CD90 is often used in combination with CD29, CD44, and CD45 to label stem cells. It is expressed in the human body in neurons and HSCs. It is also considered to be the main marker of HSC pluripotency consistent with CD34 (Craig et al., 1993; Mestas and Hughes, 2004; Majeti et al., 2007; Boitano et al., 2010). In addition, CD90 is a marker of other types of stem cells, such as MSCs, liver stem cells (Masson et al., 2006), epidermal stem cells (Nakamura et al., 2006), and endometrial progenitor/stem cells (Gargett, 2006). The present study demonstrated that rat PFCs highly expressed CD90, indicating that these cells were likely to be stem cells. CD29 is a cell surface receptor involved in cell adhesion and recognition in various processes, including embryogenesis, hemostasis, tissue repair, immune response, and metastatic spread of tumor cells (Burridge and Chrzanowska-Wodnicka, 1996). The PFCs isolated and extracted in the present study highly expressed CD29, suggesting that integrin may act as a bidirectional transmission signal between the extracellular matrix and the cytoplasmic domain and participate in the metabolism of macromolecules in the pericardial fluid. CD44 is a receptor for hyaluronic acid, a cellular protein mainly expressed in immune cells and expressed in blood, epithelial, and endothelial cells and cartilages (Patouraux et al., 2017). The CD45 molecule is expressed in all leukocytes and is called leukocyte common antigen. It is a type of transmembrane protein with a similar structure and large molecular weight. It is important for maturation, function regulation, and signal transmission. It is generally used as a marker for bone marrow cells. In the present study, the expression of CD45 in PFCs was negative, suggesting that these cells were not derived from bone marrow.

The source of PFCs is unclear. PFCs may be derived from mesothelial cells of the pericardium or from tissues other than the pericardium passing through the mesothelial cells into the pericardial fluid. We proposed an alternative cell source and the biological meaning of these cells. Our results demonstrated that PFCs express the multipotency markers CD29 CD90. After induced, PFCs has higher osteogenic but lower adipogenic potential. Meanwhile, in the process of inducing cardiac-like cells *in vitro*, pericardial fluid-derived cells apparently lacked contractile function *in vitro*. Cellular fluid is a stable biological fluid with low clearance rate and a reservoir of biologically active substances that regulate heart function. Therefore, it should have a certain importance on the diagnosis, prognosis, and treatment of heart or pericardial diseases. However, due to the relatively difficult collection of cell fluids and the ethical limitations of samples from healthy individuals, routine research and studies like those of other biological fluids (e.g., serum, plasma, saliva, urine, and joint fluid) have not been performed. Examination may be one of the reasons behind the low number of researches on the cellular composition of pericardial fluid.

## Data Availability Statement

The original contributions presented in the study are included in the article/Supplementary Material, further inquiries can be directed to the corresponding author/s.

## Ethics Statement

The animal study was reviewed and approved by the Ethics Committee of Xinxiang Medical University.

## Author Contributions

YS, ZG, and ZL conceived and designed the study. YS and YW performed the experiments. YS wrote the manuscript. ZG, ZL, and YW reviewed and edited the manuscript. All authors read and approved the manuscript.

## Conflict of Interest

The authors declare that the research was conducted in the absence of any commercial or financial relationships that could be construed as a potential conflict of interest.
