# Supplementary material for: Isolation and Multiple Differentiation of Rat Pericardial Fluid Cells
Source: Front Cell Dev Biol. 2021 Feb 11;9:614826. doi: 10.3389/fcell.2021.614826 (PMC7905039; doi:10.3389/fcell.2021.614826)
Supplement: Supplementary file 1 [file Data_Sheet_1.PDF]

Supplemental data

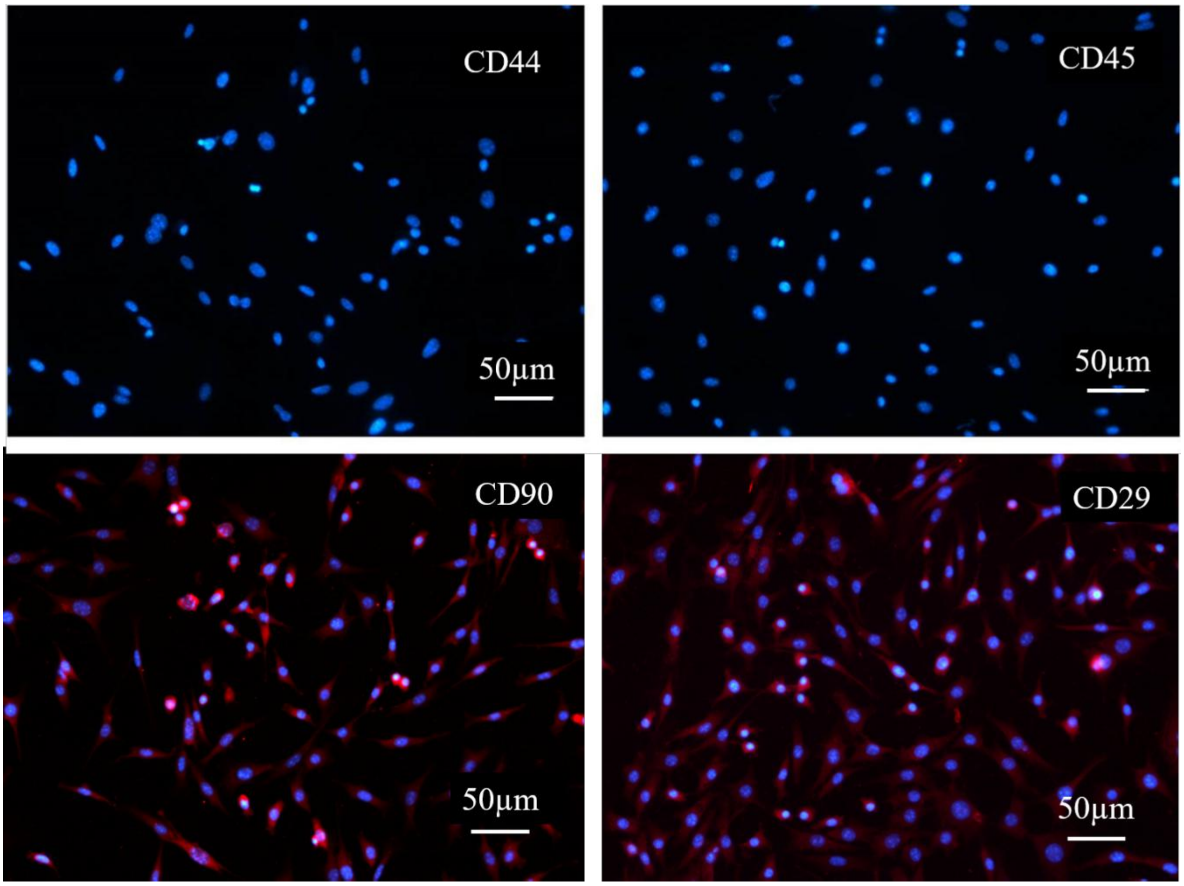

Supplementary Fig. 1 Representative images show positive expression of CD29, CD90 and negative expression of CD44, CD45. Nuclei are shown blue by DAPI. Scale bar: 50μm.

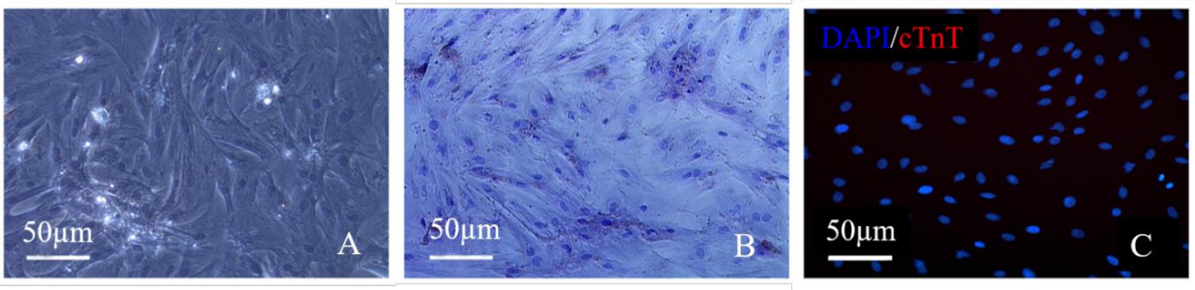

Supplementary Fig. 2 Negative control show differentiation of PFCs into osteogenic, adipogenic, and cardiomyocytes lineages. Scale bar : 50μm
